# Primary Spontaneous Orbital Hemorrhage with Secondary Orbital Roof Blow-Out Fracture: A Case Report

**DOI:** 10.3390/reports9030247

**Published:** 2026-07-31

**Authors:** Nagi A. Massoud, Mohamed Mahmoud Bakr, Abdulrahman H. Alashkar, Mohamed Ghazala

**Affiliations:** 1 Dr. Sulaiman Al-Habib Medical Group, Department of Surgery, Buraidah 51431, Qassim, Saudi Arabia; 2 Dr. Sulaiman Al-Habib Medical Group, Department of Medicine, Buraidah 51431, Qassim, Saudi Arabia; 3 Sulaiman AlRajhi University, College of Medicine, Department of Clinical Sciences, AlBukayriyah 51941, Qassim, Saudi Arabia

**Keywords:** orbital hemorrhage, orbital blowout fracture, craniotomy, case report

## Abstract

**Background and Clinical Significance**: Periorbital ecchymosis, or “raccoon eyes,” often indicates a basilar skull fracture. When trauma is absent, clinicians should consider other causes, such as Valsalva maneuvers, bleeding disorders, or systemic inflammation. Spontaneous orbital hemorrhage is rare, and its appearance as a mass causing a secondary orbital roof blow-out fracture is even more unusual. This report presents a unique case of primary spontaneous orbital hemorrhage (PSOH) leading to a secondary orbital blow-out fracture; **Case Presentation**: A 60-year-old man with a history of ulcerative colitis (UC) presented with acute-onset, non-traumatic, and bilateral periorbital bruising, swelling, and right-sided vision loss. Computed tomography (CT) showed a mass lesion in the right orbit with an associated orbital roof fracture. Following excision, histological examination showed extravasated blood. Additional laboratory work-up helped rule out possible hematologic and inflammatory etiologies, confirming a diagnosis of PSOH with a secondary orbital roof fracture; **Conclusions**: This case demonstrates that PSOH can generate sufficient intra-orbital pressure (IOP) to cause a secondary blow-out fracture, a phenomenon not previously reported in the literature. Furthermore, it emphasizes the need for a systematic diagnostic approach to exclude potential systemic etiologies in patients presenting with spontaneous periorbital ecchymosis. In addition, this case highlights the critical importance of rapid decompression in managing orbital compartment syndrome (OCS) to prevent permanent visual loss.

## 1. Introduction and Clinical Significance

Periorbital ecchymosis, frequently referred to as “raccoon eyes,” is a clinical sign classically associated with trauma and basilar skull fractures [1]. However, when this presentation occurs in the absence of trauma, it necessitates a systematic evaluation of a broad range of etiologies. Moreover, the concurrent occurrence of a non-traumatic orbital blow-out fracture is exceptionally rare. Furthermore, a history of systemic inflammatory conditions, such as ulcerative colitis (UC), can expand the differential diagnosis to include inflammatory etiologies, such as amyloidosis or IgG4-related orbital disease (IgG4-ROD) [2,3].

This case report details a 60-year-old male with a history of UC who presented with bilateral raccoon eyes and acute right-sided vision loss. The subsequent diagnosis of primary spontaneous orbital hemorrhage (PSOH), so named due to the absence of an identifiable trigger or secondary disease, resulted in a secondary orbital roof blow-out fracture. Here, we present this case and discuss the differential diagnoses we considered before making a final diagnosis of PSOH.

## 2. Case Presentation

A 60-year-old male with a history of UC presented to our emergency department with acute-onset bilateral periorbital bruising and right-sided loss of vision. He reported a sudden feeling of right-sided retro-orbital pressure and frontal headache that started about 12 h prior to his presentation to our emergency department. His symptoms progressed rapidly, and the onset of vision loss occurred roughly 7 h prior to presenting to us. He denied any trauma, diving, or maneuvers that could increase intra-orbital pressure (IOP), such as coughing, sneezing, nose blowing, heavy lifting, or straining. Furthermore, he denied a history of similar episodes. Systemic history-taking revealed no additional symptoms during or preceding the current presentation. For instance, he denied any systemic symptoms, including fever, fatigue, arthralgias, and myalgias. Moreover, he reported no skin rash or bruising, palpitations, numbness, change in bowel habit, or prior spontaneous bleeding event.

His past medical history was remarkable for UC that had been in remission for almost a year. His medications included metformin and rosuvastatin. He had no history of hypertension and no personal or family history of bleeding diathesis. He reported no history of parasomnias or sleep apnea.

**Figure 1:**
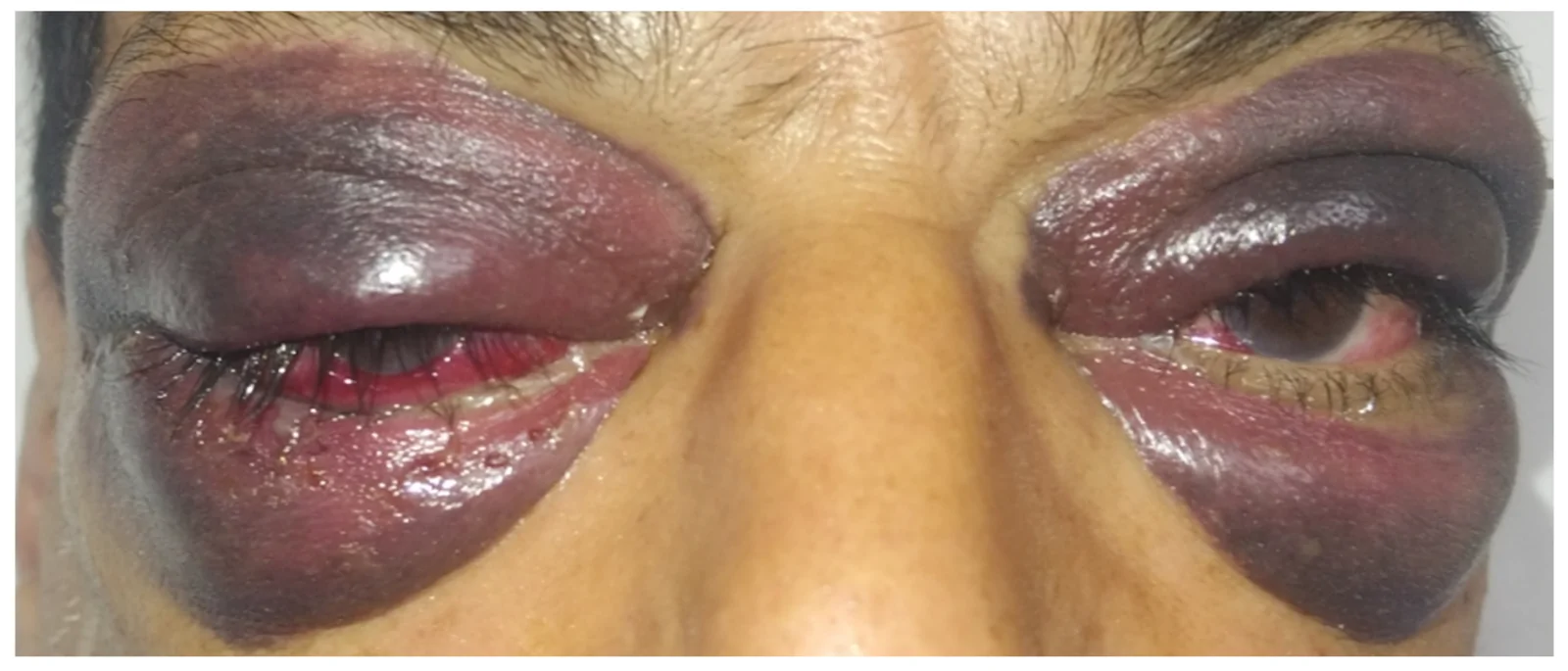
The ocular and periocular manifestations of the patient’s presentation. It shows bilateral marked periorbital ecchymosis and swelling with right-sided conjunctival chemosis and proptosis (proptosis cannot be clearly observed in this view).

On physical examination, he was fully conscious and oriented, with vital signs within normal limits. Local examination revealed bilateral periorbital ecchymosis and swelling with bilateral conjunctival chemosis and right-sided proptosis (Figure 1). He had light perception only in the right eye with 20/20 visual acuity in the left eye. He had a right-sided relative afferent pupillary defect but normal extraocular eye movements. Fundoscopy was unremarkable bilaterally. Systemic examination revealed no abnormality. Notably, he had no lymphadenopathy and no skin rash or bruising.

Blood was drawn for baseline investigations, and these investigations included a complete blood count and C-reactive protein (CRP), venous lactate, electrolytes, prothrombin time (PT), plasma thromboplastin time (PTT), and liver and kidney function tests. All test results were within normal limits apart from a mildly elevated CRP (Table 1).

**Figure 2:**
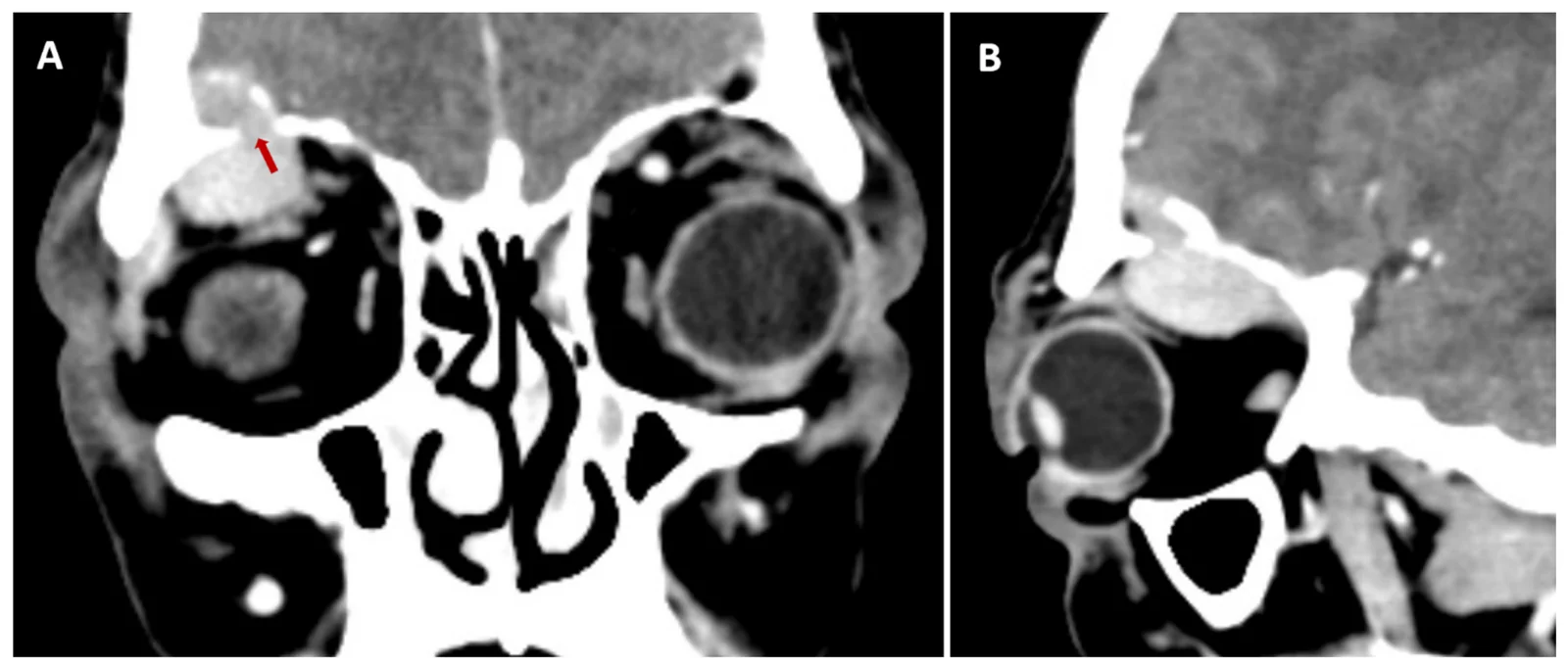
Preoperative CT images. (**A**) is a coronal view showing a right-sided orbital lesion with an overlying osseous defect (red arrow). The lesion is noted to extend into the anterior skull base. (**B**) is a sagittal view showing the location of the osseous defect in relation to the anterior skull base.

Computed tomography (CT) scan of the brain showed a superolateral hyperdense lesion in the right orbital cavity with intracranial extension through an orbital roof fracture (Figure 2). Gadolinium-enhanced magnetic resonance imaging of the brain revealed the mass lesion to be hypointense on T1-weighted sequence, hyperintense on T2-weighted sequence, and non-enhancing. It also demonstrated mild pachymeningeal enhancement at the right frontotemporal region. MR venography showed no abnormality.

**Figure 3:**
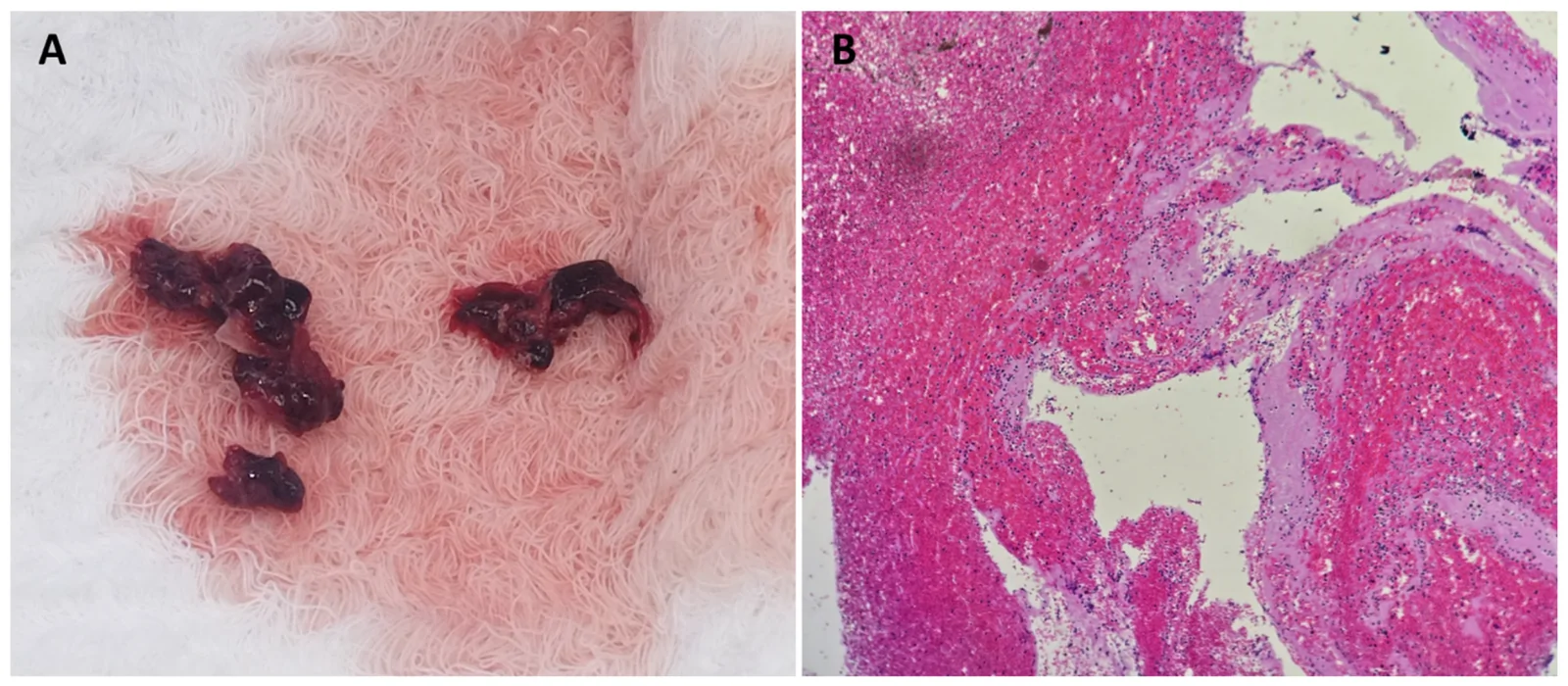
(**A**). The tissue pieces excised from the patient’s right orbit. (**B**). A histological slide showing extravasated blood and scattered fibrin with no evidence of neoplastic process (magnification 40x).

An urgent right zygomatico-orbital craniotomy was performed. The mass was excised in pieces (Figure 3A), and the orbital roof was reconstructed with a titanium mesh. Histological examination showed the excised lesion to be composed of extravasated blood and scattered fibrin, with no evidence of vascular malformation or neoplastic process (Figure 3B). Additional investigations were requested postoperatively, including urine and serum electrophoresis, urine albumin/creatinine ratio, factor XIII antigen level, and serum free light chain level. All investigation results were unremarkable. A diagnosis of primary spontaneous orbital hemorrhage (PSOH) with secondary orbital roof blow-out fracture was made. The patient was discharged on the fifth postoperative day in a stable condition. One month postoperatively, his periorbital ecchymosis was almost completely resolved with no proptosis. He had normal pupillary reflexes bilaterally. His right eye showed partial visual recovery, but it remained severely impaired with visual acuity of 20/400.

## 3. Discussion

Periorbital ecchymosis, commonly known as “raccoon eyes,” is classically associated with basilar skull fractures [1]. However, in the absence of trauma, a diverse range of etiologies may be considered, requiring systematic evaluation to establish the diagnosis. Three aspects in this case might have expanded the differential diagnosis and precluded direct spot diagnosis of orbital hemorrhage. First, he had no readily identifiable trigger, such as trauma, Valsalva-like maneuvers, or a bleeding diathesis. The second is the presence of a hemorrhagic “lesion” and an orbital roof defect/fracture. The final aspect is the background history of UC.

Increased venous pressure induced by severe Valsalva-like maneuvers, such as severe vomiting and coughing, represents a recognized mechanism for orbital hemorrhage. This is especially true in patients with a bleeding diathesis or orbital infection [4]. Such maneuvers increase the intrathoracic pressure. This pressure is transmitted throughout the venous system, including the orbital vasculature [5]. The orbital blood vessels are particularly vulnerable to such pressure increases because they exist within a rigid bony compartment with limited compliance [6]. When venous outflow pressure increases, the vessels within the orbit expand and become susceptible to rupture. Prior orbital trauma or surgery might make its vasculature even more fragile [7]. Our patient had no history of trauma or surgery. He also denied any Valsalva-like maneuvers during or preceding the onset of his symptoms. We also explored whether the patient had sleep disorders, such as parasomnias or sleep apnea, that could be associated with hidden events, explaining the hemorrhage (for example, trauma during sleepwalking). This was also negative in our patient.

Another possible trigger we considered is a bleeding diathesis. However, our patient is a 60-year-old with no personal or family history of bleeding disorders and no bleeding events in the past. Moreover, his platelet count, PT, and PTT were within normal limits. Following our hematologist’s recommendation, we considered factor XIII deficiency, which can present with spontaneous bleeding and normal routine screening assays [8]. Nevertheless, the quantitative FXIII activity assay result was within the normal limits.

Another perplexing feature in our case was the presence of an orbital “lesion” rather than diffuse hemorrhage, which was also associated with an orbital roof fracture. Non-traumatic orbital blow-out fractures are rare. They have been documented in a few case reports and in association with Valsalva-like maneuvers [9]. Furthermore, a few cases of acute spontaneous orbital hemorrhages appearing as mass “lesions” have been reported in the literature [10]. However, to our knowledge, such spontaneous orbital hemorrhages have not been previously reported to cause orbital blow-out fractures.

In addition to the absence of possible triggers and the presence of unexpected imaging findings, the history of UC further expanded the differential diagnosis to include inflammatory etiologies, namely, amyloidosis and IgG4-related orbital disease (IgG4-ROD) [2,3]. Amyloidosis is a heterogeneous group of rare diseases characterized by the abnormal misfolding and extracellular deposition of fibrillar proteins in various tissues. It can be subdivided into primary and secondary forms, with the secondary form being relevant to inflammatory bowel diseases, including UC, and resulting from the prolonged overproduction of serum amyloid-A protein in response to chronic inflammation [11]. Orbital involvement in amyloidosis, particularly AL (primary) amyloidosis, can present as discrete mass lesions. Moreover, periorbital purpura (i.e., raccoon eye appearance) in amyloidosis is well documented in the medical literature. A comprehensive case series analysis identified 28 patients with AL amyloidosis characterized by this appearance [12]. Individual case reports further illustrate the acute presentation of this phenomenon. One case described a 65-year-old man with proteinuria, poor appetite, and weight loss who developed left periorbital ecchymosis [13]. Another notable case involved a 64-year-old woman who developed periorbital purpura acutely within 24 h following a kidney biopsy [14]. Nevertheless, both these patients had renal and systemic involvement prior to their ocular presentation. However, our patient did not have any extraocular involvement; in particular, he had no evidence of renal involvement (proteinuria). Moreover, his UC had been in remission for a year prior to his presentation. Therefore, the patient’s history of UC was likely coincidental.

Another inflammatory etiology that we considered was IgG4-ROD. IgG4-related disease (IgG4-RD) is a systemic fibroinflammatory condition. It can affect nearly any organ system, including the orbital and periorbital tissues [15]. IgG4-ROD represents a well-recognized site-specific manifestation of systemic IgG4-RD, with orbital involvement occurring in approximately 17% to 74% of IgG4-RD patients, depending on the cohort studied. The most common manifestations of IgG4-ROD are dacryoadenitis and bilateral eyelid edema [16]. Optic neuropathy is a potentially sight-threatening complication of IgG4-ROD. This complication can result from mass effect, infraorbital nerve enlargement, or optic nerve perineuritis [17]. Several clinical aspects in our case argued against it: the hyperacute presentation (the patient’s symptoms developed over hours) and the absence of systemic and lacrimal involvement.

In our case, all blood investigations, including coagulation profile, complete blood count, inflammatory markers, plasma/urine electrophoresis, serum light chains, and IgG4 level, were unremarkable. However, it was the tissue-based assessment that really ruled out neoplastic and inflammatory etiologies and confirmed the final diagnosis of primary spontaneous orbital hemorrhage (PSOH) with secondary orbital roof blow-out fracture. To our knowledge, this clinical presentation has not been reported previously. Notably, McNab AA proposed using the term “non-traumatic orbital hemorrhage” instead of other descriptions, such as idiopathic or spontaneous hemorrhage. He argued that such cases have an underlying cause, such as increased intra-orbital pressure or a bleeding diathesis [18]. However, this is not true in our case. Therefore, we used the descriptions of primary, due to the absence of secondary disease (bleeding diathesis, neoplastic, or inflammatory), and spontaneous, due to the absence of a trigger (trauma or Valsalva-like maneuvers).

The way we approached this case was to urgently perform a zygomatico-orbital craniotomy to decompress the optic nerve, as well as excising the lesion for histological assessment. Notably, the acute vision loss was probably due to increased IOP, as the lesion was not directly compressing the optic nerve. In other words, it was due to orbital compartment syndrome (OCS).

Normal IOP is less than 20 mmHg, and acute increases can lead to dramatic decreases in perfusion, with subsequent ischemia comparable to that seen in compartment syndromes in other body regions [19]. A brief, 60–100 min rise in IOP can lead to permanent visual sequelae [20]. In a major retrospective analysis of orbital hemorrhage with OCS, the median time between diagnosis and management was significantly shorter in patients who achieved visual recovery compared with those who developed permanent blindness or sequelae, i.e., 6 h versus 12 h, respectively [21]. This emphasizes that while irreversible damage can take 60–100 min to occur, the critical management window is longer, with superior outcomes when decompression occurs within a 6 h window. However, visual recovery is possible even if decompression is performed beyond 6 h, suggesting that the timing effect on outcome is not all-or-nothing but rather exists on a continuum, with better outcomes associated with earlier intervention [21]. Therefore, we must say that our patient should have undergone lateral canthotomy in the emergency department to acutely decompress the orbit, even though his presentation was delayed for over seven hours since the onset of vision loss [21, 22].

## 4. Conclusions

This case highlights an unusual presentation of PSOH resulting in a secondary orbital roof blow-out fracture. While non-traumatic periorbital ecchymosis typically suggests a systemic issue or Valsalva-induced pressure, it can result from spontaneous orbital hemorrhage with no underlying systemic disease. Furthermore, this hemorrhage can generate sufficient IOP to cause a bony fracture.

In this case, a wide differential diagnosis was considered based on the patient’s clinical presentation and medical history, highlighting the importance of a tailored diagnostic approach. Finally, this case discussed the importance of rapid decompression in managing OCS to prevent permanent visual loss, and clinicians should consider immediate bedside intervention, such as lateral canthotomy, even in cases of delayed presentation.

## Figures and Tables

**Table 1:** Results of select investigations.

Test	Result	Reference Range
White blood cell count	10,900/μL	4000–11,000/μL
Platelet count	271,000/μL	150,000–450,000/μL
Hemoglobin	13 g/dL	11.8–14.8 g/dL
Serum creatinine	63.53 μmol/L	49–90 μmol/L
Serum sodium	139 mmol/L	136–145 mmol/L
Serum potassium	4.1 mmol/L	3.5–5.1 mmol/L
Ionized calcium	1.14 mmol/L	1.12–1.32 mmol/L
ALT	22 IU/L	5–55 IU/L
AST	26 IU/L	5–34 IU/L
Albumin	37 g/L	32–46 g/L
PTT	28.5 s	28–40 s
PT	12.1 s	10.5–15 s
CRP	7.1 mg/L	<5 mg/L
Factor XIII antigen	74%	59–181%
IgG4	0.22 g/L	<2 g/L

Abbreviations: ALT, alanine aminotransferase; AST, aspartate aminotransferase; PTT, partial thromboplastin time; PT, prothrombin time; CRP, C-reactive protein; IgG4, immunoglobulin G4.

## Data Availability

Original data generated and analyzed during this study are included in this published article.

## Abbreviations

The following abbreviations are used in this manuscript:

PSOH	primary spontaneous orbital hemorrhage
IgG4-RD	immunoglobulin G4-related disease
UC	ulcerative colitis
IOP	intra-orbital pressure
CT	computed tomography
IgG-ROD	IgG4-related orbital disease
OCS	orbital compartment syndrome
